# Molecular detection, characterization, and cross-species circulation of feline and canine parvoviruses in Gujarat, India: Emergence of a novel canine parvovirus-2c variant and enhanced diagnostic sensitivity of quantitative polymerase chain reaction

**DOI:** 10.14202/vetworld.2026.3138-3155

**Published:** 2026-07-20

**Authors:** Fatimazohra Abdulrashidkhan Pathan, Arunkumar C. Patel, Niyati M. Rana, Sejal P. Antiya, Prakash G. Koringa, Rafiyuddin A. Mathakiya, Vipul R. Nimavat, Ankit S. Prajapati

**Affiliations:** 1 Department of Veterinary Microbiology, College of Veterinary Science and Animal Husbandry, Kamdhenu University, Anand-388001, Gujarat, India.; 2 Department of Veterinary Biotechnology, College of Veterinary Science and Animal Husbandry, Kamdhenu University, Anand-388001, Gujarat, India.; 3 Department of Veterinary Medicine, College of Veterinary Science and Animal Husbandry, Kamdhenu University, Anand-388001, Gujarat, India.

**Keywords:** canine parvovirus, cross-species transmission, feline parvovirus, molecular epidemiology, phylogenetic analysis, quantitative polymerase chain reaction, VP2 gene, viral evolution

## Abstract

**Background and Aim:**

Feline parvovirus (FPV) and canine parvovirus (CPV) are highly contagious pathogens that cause severe enteritis in companion animals, leading to considerable morbidity and mortality, particularly in young and unvaccinated animals. The continuous evolution of CPV variants and increasing evidence of cross-species transmission present major challenges for disease diagnosis, surveillance, and control. This study aimed to determine the prevalence of FPV and CPV infections among cats and dogs in Gujarat, India, compare the diagnostic performance of rapid antigen testing (RAT), conventional polymerase chain reaction (PCR), and quantitative PCR (qPCR), characterize circulating viral variants through *VP2* gene sequencing and phylogenetic analysis, and investigate evidence of cross-species circulation.

**Materials and Methods:**

A cross-sectional study was conducted between September 2024 and September 2025 using 200 fecal samples collected from 100 cats and 100 dogs presented to veterinary clinics, shelters, and rescue organizations in Gujarat, India. Samples were examined using RAT, conventional PCR targeting the *VP2* gene, and duplex probe-based qPCR to differentially detect FPV and CPV. Representative positive samples were subjected to *VP2* gene sequencing, phylogenetic analysis, and virus isolation in Crandell-Rees feline kidney cell cultures to evaluate viral replication. Epidemiological characteristics, including species, age, breed, vaccination status, and clinical presentation, were also assessed.

**Results:**

RAT detected FPV and CPV in 10 (5.0%) and 80 (40.0%) samples, respectively, whereas conventional PCR identified 39 (19.5%) parvovirus-positive samples. Duplex qPCR demonstrated markedly superior sensitivity, detecting 124 (62.0%) positive samples, comprising 27 FPV and 97 CPV infections. Cross-species circulation was demonstrated by detection of CPV in 12% of cats and FPV in 1% of dogs. Sequencing of the *VP2* gene revealed genetically conserved FPV isolates and predominance of CPV-2c among canine isolates, including a novel CPV-2c variant carrying unique amino acid substitutions suggestive of ongoing viral evolution. Virus isolation successfully confirmed replication in four of nine inoculated samples, producing characteristic cytopathic effects in Crandell-Rees feline kidney cells.

**Conclusion:**

FPV and CPV co-circulate among companion animals in Gujarat, with molecular evidence of cross-species transmission and predominance of CPV-2c variants. Duplex qPCR substantially outperformed RAT and conventional PCR, supporting its routine application for accurate diagnosis, epidemiological surveillance, and early detection of emerging parvovirus variants. Continuous molecular surveillance combined with sustained vaccination programs is essential to limit viral dissemination and improve control of parvoviral infections.

## INTRODUCTION

Feline parvovirus (FPV) and canine parvovirus (CPV) are among the most important viral pathogens affecting companion animals worldwide. Both viruses belong to the genus *Protoparvovirus* within the family *Parvoviridae* and primarily infect rapidly dividing cells of the intestinal epithelium, bone marrow, and lymphoid tissues. Infection commonly results in acute hemorrhagic enteritis, severe leukopenia, dehydration, and high mortality, particularly in young and unvaccinated animals [1, 2]. Although effective vaccines are available, parvoviral enteritis continues to occur globally due to incomplete vaccination, interference by maternally derived antibodies, vaccine failure, and the ongoing emergence of novel viral variants [2, 3].

FPV shares a high degree of genetic and antigenic similarity with CPV, which is believed to have originated from FPV through host-range mutations in the late 1970s [4]. Subsequent viral evolution resulted in the emergence of CPV-2a, CPV-2b, and CPV-2c variants, several of which have been identified in feline hosts and produce clinical manifestations indistinguishable from classical feline panleukopenia [5, 6]. Host adaptation is primarily associated with mutations in the *VP2* capsid protein, which governs viral antigenicity, receptor binding, and host tropism [7, 8]. The *VP2* gene remains the principal target for molecular epidemiological investigations because sequence variations can influence viral virulence, antigenic characteristics, host adaptation, and vaccine efficacy [9]. Molecular diagnostic techniques such as PCR, qPCR, and *VP2* gene sequencing have therefore become indispensable tools for accurate diagnosis, differentiation of circulating variants, molecular surveillance, and monitoring of parvovirus evolution [10, 11].

Companion cats and dogs in India frequently coexist in households, shelters, rescue centers, breeding facilities, and free-roaming populations, creating favorable conditions for interspecies exposure and transmission of parvoviruses. Although several investigations have reported the occurrence of FPV and CPV infections in different regions of India, comprehensive molecular epidemiological information from Western India, particularly Gujarat, remains limited. Furthermore, previous studies have largely focused on either feline or canine populations, with relatively few studies simultaneously evaluating both host species to determine the extent of cross-species circulation. In addition, comparative assessments of the diagnostic performance of rapid antigen testing (RAT), conventional PCR, and qPCR under field conditions remain scarce. Information regarding the molecular characteristics of circulating *VP2* variants, their phylogenetic relationships, and the emergence of novel CPV variants in this region is also insufficient. These knowledge gaps limit the understanding of regional viral evolution, transmission dynamics, diagnostic accuracy, and the effectiveness of existing surveillance and vaccination strategies. Therefore, updated molecular epidemiological data are essential to strengthen disease surveillance, improve diagnostic approaches, optimize vaccination programs, and support evidence-based control strategies for parvoviral infections in companion animals.

The present study aimed to determine the prevalence of FPV and CPV infections among companion cats and dogs in Gujarat, India, using molecular and immunological diagnostic approaches. Specifically, the study compared the diagnostic performance of RAT, conventional PCR, and probe-based qPCR for detecting parvoviral infections; characterized circulating viral variants through *VP2* gene sequencing and phylogenetic analysis; investigated evidence of cross-species transmission between feline and canine hosts; evaluated the successful isolation of circulating viruses in cell culture; and assessed epidemiological characteristics associated with infection, including species, age, breed, vaccination status, and clinical presentation. Collectively, these findings were intended to provide updated regional molecular epidemiological data to support improved diagnosis, surveillance, and control of FPV and CPV infections in companion animals.

## MATERIALS AND METHODS

### Ethical approval

The present study involved the non-invasive collection of fecal samples and rectal swabs from companion cats and dogs presented to veterinary clinics, rescue organizations, and animal shelters. Sample collection was performed by licensed veterinarians following standard veterinary clinical procedures to minimize animal stress and discomfort. Written informed consent was obtained from all animal owners or authorized caretakers before sample collection. No animals were subjected to experimental infection, invasive procedures, or harmful interventions specifically for the purpose of this study. All sampling procedures complied with institutional guidelines for animal welfare and ethical research involving animals. The research does not require approval from the Institutional Animal Ethics Committee of the College of Veterinary Science and Animal Husbandry, Kamdhenu University, Anand, Gujarat, India.

### Study period and location

This cross-sectional study was conducted between September 2024 and September 2025 in and around Anand District, Gujarat, India. Laboratory investigations, including molecular detection, virus isolation, sequencing, and phylogenetic analyses, were performed at the Department of Veterinary Biotechnology, College of Veterinary Science and Animal Husbandry, Kamdhenu University, Anand, Gujarat, India.

### Study design

A cross-sectional epidemiological study was designed to determine the prevalence, molecular characteristics, and cross-species circulation of FPV and CPV among companion animals. The study further compared the diagnostic performance of RAT, conventional PCR, and qPCR and evaluated the molecular diversity of circulating viruses through *VP2* gene sequencing and phylogenetic analysis.

### Sample collection

A total of 200 fecal samples were collected from 100 cats and 100 dogs presented to the Veterinary Clinical Complex, College of Veterinary Science and Animal Husbandry, Anand, Gujarat, India, as well as from private veterinary clinics, rescue organizations, and animal shelters in the surrounding region. Samples were obtained from animals exhibiting clinical signs suggestive of gastroenteritis, including diarrhea, vomiting, dehydration, anorexia, and abdominal discomfort, as well as from apparently healthy animals for comparative analysis.

Freshly voided feces or rectal swabs were collected aseptically using sterile screw-cap swabs (HiMedia Laboratories Pvt. Ltd., Mumbai, India). Swabs were either collected dry or pre-moistened with Hanks' Balanced Salt Solution to maintain viral stability. Each sample was assigned a unique identification code according to the host species. Samples were transported to the laboratory on ice in insulated containers and stored at 4°C for short-term processing or at −20°C for long-term storage until further analysis. Apparently healthy animals were defined as those not exhibiting clinical signs of gastrointestinal disease at the time of sampling.

### DNA extraction

Viral DNA was extracted using the QIAamp DNA Mini Kit (Qiagen, Hilden, Germany) according to the manufacturer's instructions. Briefly, 200 µL of a 10% (w/v) fecal suspension prepared in phosphate-buffered saline (pH 7.4) was used for extraction. Samples were lysed using proteinase K and Buffer AL, followed by column-based purification. DNA was eluted in 50 µL of elution buffer and stored at −20°C until analysis.

DNA concentration and purity were determined using a NanoDrop spectrophotometer (Thermo Fisher Scientific, Waltham, MA, USA). Extraction controls were included throughout the procedure to monitor potential contamination and ensure extraction quality. Samples with A260/A280 ratios between 1.8 and 2.0 were considered acceptable for downstream molecular analyses.

### PCR detection of FPV and CPV

Conventional PCR targeting a conserved region of the *VP2* gene was performed using primers described by Carreno *et al.* [12]. Owing to the high sequence similarity between FPV and CPV, these primers amplified parvoviral DNA from both feline and canine samples; however, differentiation between the two viruses was not possible using conventional PCR alone.

Each PCR reaction was prepared in a total volume of 25 µL containing 12.5 µL of 2× PCR Master Mix (Takara Bio Inc., Kusatsu, Japan), 10 pmol of each primer, 2 µL of template DNA, and nuclease-free water to the final reaction volume.

Thermal cycling conditions consisted of an initial denaturation at 95°C for 5 min, followed by 35 cycles of denaturation at 95°C for 30 s, annealing at 55°C for 30 s, and extension at 72°C for 1 min, with a final extension at 72°C for 10 min.

PCR products were resolved on a 1% agarose gel prepared in Tris-borate-EDTA buffer at 90 V for 50 min, stained with ethidium bromide, and visualized under ultraviolet illumination using a gel documentation system (Bio-Rad Laboratories, Hercules, CA, USA).

Positive controls, negative controls, and no-template controls (NTC) were included in every PCR run to ensure assay reliability. Previously validated primers targeting conserved regions of the *VP2* gene were used to ensure assay specificity. The primer and probe sequences used in this study are presented in Table 1.

### Quantitative PCR for virus differentiation

The duplex probe-based qPCR assay was performed as described by Decaro *et al.* [13] for the simultaneous detection and differentiation of FPV and CPV. The assay targeted the *VP2* gene using virus-specific hydrolysis probes labeled with FAM for CPV and VIC for FPV.

Each reaction was prepared in a final volume of 20 µL containing 10 µL of 2× TaqMan™ Universal PCR Master Mix (Thermo Fisher Scientific), 0.4 µM of each primer, 0.2 µM of each probe, and 2 µL of template DNA.

Amplification was carried out using a QuantStudio Real-Time PCR System (Thermo Fisher Scientific) under the following cycling conditions: initial activation at 95°C for 10 min, followed by 40 cycles of denaturation at 95°C for 15 s and annealing/extension at 50°C for 60 s.

Fluorescence signals were recorded during each amplification cycle, and cycle threshold (Ct) values ≤35 were considered positive. All reactions were performed in duplicate and included positive controls, negative controls, and NTCs. Assay performance was evaluated based on consistent amplification of positive controls and the absence of amplification in negative and NTC. Primer and probe details are provided in Table 1.

**Table 1:** Primers and probes used in the present study.

**Target virus**	**Primer/probe**	**Sequence (5′–3′)**	**Assay type**	**Amplicon size (bp)**	**Reference**
FPV (*VP2*)	FPV F	CAGGAAGATATCCAGAAGGA	PCR	681	[12]
	FPV R	GGTGCTAGTTGATATGTAATAAACA			
FPV/CPV	FPV/CPV-F	ACAAGATAAAAGACGTGGTGTAACTCAA	Probe-based qPCR	83	[13]
	FPV/CPV-R	CAACCTCAGCTGGTCTCATAATAGT			
	FPV probe	VIC-ATGGGAAATACAGACTATAT-BHQ			
	CPV probe	FAM-ATGGGAAATACAAACTATAT-BHQ			

FPV = Feline parvovirus, CPV = Canine parvovirus.

### Virus isolation in cell culture

Virus isolation was attempted using the Crandell-Rees feline kidney (CRFK) cell line obtained from the Indian Veterinary Research Institute, Bengaluru, India. Cells were maintained in Dulbecco's Modified Eagle Medium supplemented with 10% fetal bovine serum and 1% antibiotic mixture containing penicillin (100 U/mL), streptomycin (100 µg/mL), and amphotericin B (0.25 µg/mL). Cell cultures were incubated at 37°C and routinely monitored to confirm the absence of mycoplasma contamination.

Before inoculation, fecal supernatants were filtered through 0.22-µm syringe filters to remove bacterial contaminants. Approximately 200 µL of clarified inoculum was added to each well. Blind passages were performed every 3–4 days by transferring 200 µL of culture supernatant onto fresh CRFK monolayers. All procedures were conducted under biosafety level 2 conditions.

Following adsorption at 37°C for 1 h with intermittent rocking, maintenance medium containing 2% fetal bovine serum was added. Cell cultures were examined daily for cytopathic effects (CPE) for up to 7 days. Samples that failed to produce CPE were subjected to up to 3 blind passages to improve viral recovery.

### Sequencing and molecular characterization

PCR products were purified using a commercial PCR purification kit and subjected to Sanger sequencing using an ABI 3730 Genetic Analyzer (Applied Biosystems, Foster City, CA, USA). Sequencing was performed with forward primers, and sequence quality was assessed prior to downstream analyses.

Sequence editing and assembly were performed using BioEdit software. Similarity searches were conducted using the Basic Local Alignment Search Tool available through the National Center for Biotechnology Information (NCBI).

Phylogenetic analysis was performed using Molecular Evolutionary Genetics Analysis version 12 software. The evolutionary history was inferred using the Maximum Likelihood method based on the Tamura three-parameter model with 1,000 bootstrap replicates. Because only partial *VP2* gene sequences were obtained, complete genetic characterization and definitive subtype differentiation of the circulating strains were limited.

### Statistical analysis

Data generated in the present study were analyzed using descriptive statistics. The prevalence of FPV and CPV infections was calculated as the percentage of positive samples among all samples examined. Because of the observational nature of the study and the absence of complete epidemiological data for all investigated variables, advanced statistical analyses to determine associations or risk factors were not performed.

## RESULTS

### Study population and epidemiological characteristics

A total of 200 fecal samples were collected from companion animals in and around Anand District, Gujarat, India, comprising 100 cat and 100 dog samples. Among the sampled animals, 117 (58.5%) showed clinical signs consistent with gastroenteritis, including diarrhea, vomiting, dehydration, anorexia, and abdominal discomfort, whereas 83 (41.5%) were apparently healthy at the time of sampling. Samples were obtained from the Veterinary Clinical Complex, private veterinary clinics, rescue organizations, and shelters, representing a heterogeneous companion animal population.

Animals aged 3–6 months constituted the majority of positive cases. The study population included 113 males (54 cats and 59 dogs) and 87 females (46 cats and 41 dogs). Breed distribution showed that non-descript and Persian cats were similarly represented, whereas Labrador Retrievers constituted the largest proportion among dogs, followed by German Shepherds.

Vaccination history showed that 62 animals were fully vaccinated, 57 were partially vaccinated, and 81 were unvaccinated. FPV detection was lower among fully vaccinated cats, whereas CPV infection was observed in dogs irrespective of vaccination status. Seasonal distribution showed higher positivity during summer and winter, suggesting potential environmental influences on viral persistence and transmission.

RAT detected FPV antigen in 10 (5.0%) samples and CPV antigen in 80 (40.0%) samples. Conventional PCR targeting the *VP2* gene detected 39 positive samples (19.5%), comprising 11 feline and 28 canine samples. In contrast, probe-based qPCR detected 124 positive animals (62.0%), including 27 FPV-positive and 97 CPV-positive cases, demonstrating substantially higher sensitivity.

### Molecular detection of FPV and CPV

**Conventional PCR:** Amplification targeting the *VP2* gene produced the expected 681 bp amplicon, confirming the presence of parvoviral DNA in fecal samples. Of the 200 samples screened, 39 (19.5%) tested positive, including 11 cats (11%) and 28 dogs (28%).

**Figure 1:**
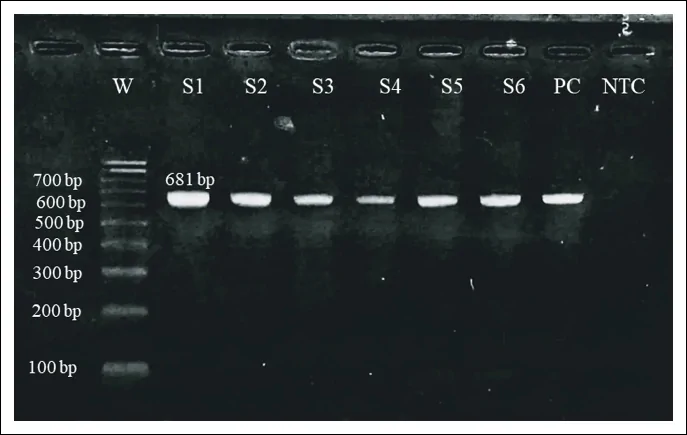
Agarose gel electrophoresis image of *VP2* gene polymerase chain reaction-amplified products of feline parvovirus. Lane W: 100 bp DNA ladder (Catalog #SM0243, Applied Biosystems™, Thermo Fisher Scientific Inc.); lanes S1–S6: Positive samples showing the target-specific amplicon of 681 bp; lane PC: Positive control; lane NTC: No-template control.

The primers used in this assay targeted conserved regions of the *VP2* gene and therefore detected both FPV and CPV. Consequently, although PCR confirmed the presence of parvoviral DNA, it could not differentiate between FPV and CPV infections. The amplified PCR products were resolved on 1% agarose gel, and a specific amplicon of approximately 681 bp was observed in positive samples, confirming the presence of parvoviral DNA (Figure 1).

**qPCR detection and differentiation:** A probe-based multiplex qPCR assay targeting virus-specific regions of the *VP2* gene was used to differentiate FPV and CPV infections. Among the 200 samples tested, 124 (62.0%) were positive for parvovirus, including 26 FPV-positive and 12 CPV-positive cats, and 85 CPV-positive and one FPV-positive dog.

**Figure 2:**
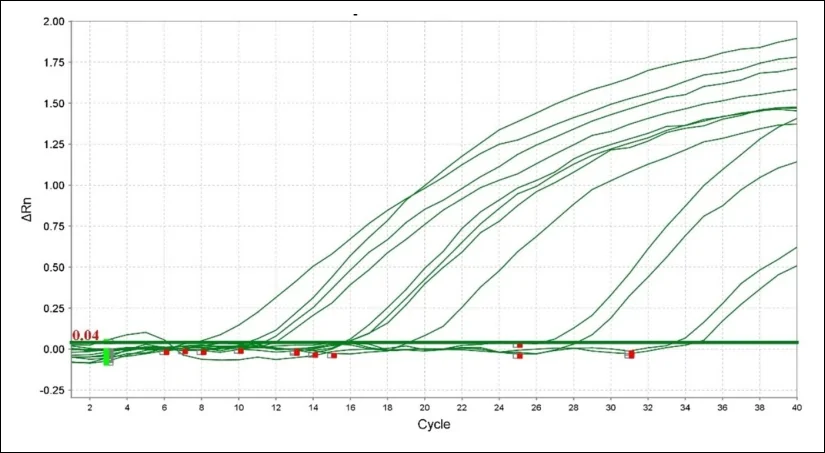
Quantitative polymerase chain reaction amplification plot for feline parvovirus.

**Figure 3:**
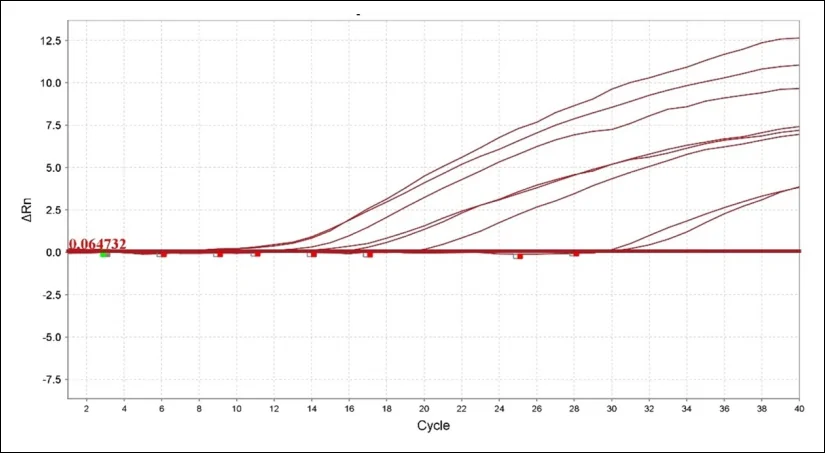
Quantitative polymerase chain reaction amplification plot for canine parvovirus.

**Figure 4:**
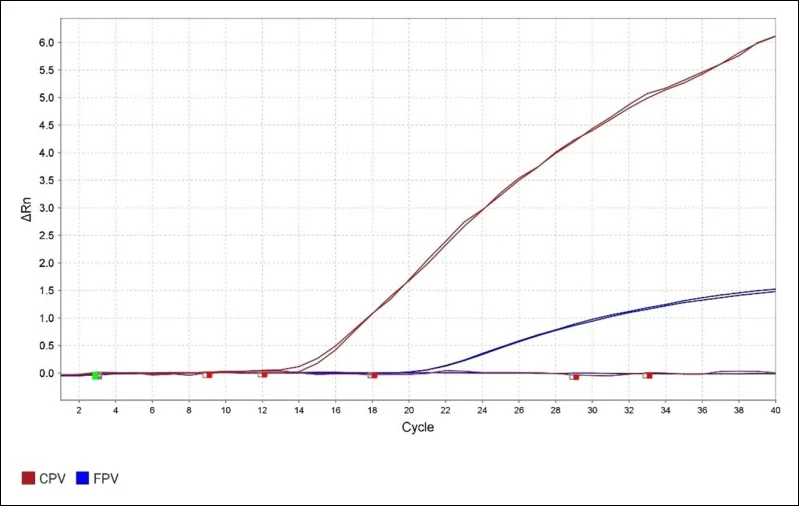
Quantitative polymerase chain reaction amplification plot for feline parvovirus and canine parvovirus.

Among feline samples, 26% were FPV-positive and 12% were CPV-positive, whereas one canine sample tested positive for FPV, indicating possible cross-species circulation of parvoviruses between feline and canine hosts. Among dogs, 85% tested positive for CPV, confirming widespread circulation within the canine population. The qPCR amplification plots for FPV, CPV, and the combined detection of FPV and CPV are presented in Figures 2, 3, and 4, respectively.

### Comparative evaluation of diagnostic methods

Comparison of diagnostic methods revealed marked differences in detection rates. RAT detected only 5% of cases, whereas conventional PCR detected 19.5%. In contrast, qPCR detected 62% of infections, demonstrating higher analytical sensitivity.

The detection pattern observed in this study was as follows:

RAT (5%) < PCR (19.5%) < qPCR (62%)

The comparative diagnostic performance of RAT, conventional PCR, and qPCR for the detection of FPV and CPV in cats and dogs is summarized in Table 2.

PCR primers described by Carreno *et al.* [12] target a conserved *VP2* region and therefore detect parvoviral DNA without differentiating FPV and CPV. Differentiation of FPV and CPV was performed using the probe-based qPCR assay described by Decaro *et al.* [26].

### Virus isolation

Virus isolation was attempted from nine molecularly confirmed parvovirus-positive samples using CRFK cell monolayers. Four samples produced characteristic CPE, including cell rounding, aggregation, granulation, and detachment, beginning between the third and fourth passages. These cultures eventually showed partial to complete destruction of the monolayer within 48–72 h.

**Figure 5:**
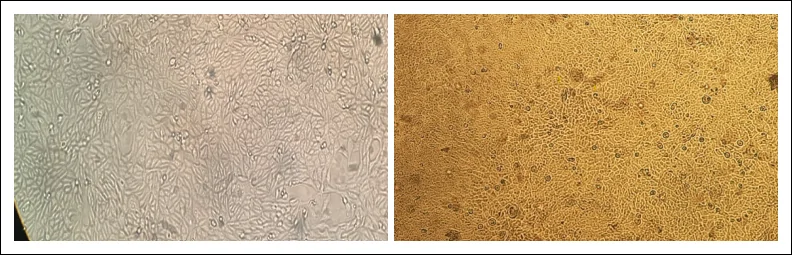
Cytopathic changes in Crandell–Rees feline kidney (CRFK) cells after parvovirus inoculation. Left: Uninfected CRFK cells showing normal confluent monolayer morphology (40×, inverted microscope). Right: CRFK cells inoculated with suspected parvovirus showing mild cytopathic changes (40×, inverted microscope).

**Figure 6:**
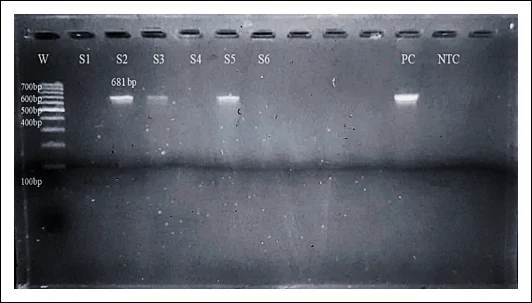
Polymerase chain reaction amplification of the *VP2* gene from infected cell culture confirming parvoviral DNA (681 bp). Lane W: 100 bp DNA ladder (Catalog #SM0243, Applied Biosystems™, Thermo Fisher Scientific Inc.); lanes S1, S4, and S6: Negative samples; lanes S2, S3, and S5: Positive samples showing the target-specific amplicon of 681 bp; lane PC: Positive control; lane NTC: No-template control.

PCR confirmation of culture supernatants produced the expected 681 bp *VP2* amplicon, verifying viral replication in infected cell cultures. Overall, 44.44% of samples were successfully adapted to CRFK cells. The CPE observed during virus isolation in CRFK cell culture is shown in Figure 5, whereas PCR amplification of the isolated virus, showing the specific *VP2* gene band, is shown in Figure 6.

**Table 2:** Comparative detection of parvoviruses by RAT, conventional PCR, and qPCR

**Species**	**RAT FPV**	**RAT CPV**	**PCR FPV/CPV**	**qPCR FPV**	**qPCR CPV**
Cats (n = 100)	8 (8%)	10 (10%)	11 (11%)	26 (26%)	12 (12%)
Dogs (n = 100)	2 (2%)	70 (70%)	28 (28%)	1 (1%)	85 (85%)
Total (n = 200)	10 (5%)	80 (40%)	39 (19.5%)	27 (13.5%)	97 (48.5%)

RAT = Rapid antigen testing, PCR = Polymerase chain reaction, qPCR = Quantitative PCR, FPV = Feline parvovirus, CPV = Canine parvovirus.

### Molecular characterization of parvovirus isolates

Sequencing of the *VP2* gene was performed on 10 representative isolates, including 3 FPV and 7 CPV samples. FPV isolates showed 99.8%–100% nucleotide identity with classical FPV strains reported globally and displayed only minor amino acid substitutions.

**Figure 7:**
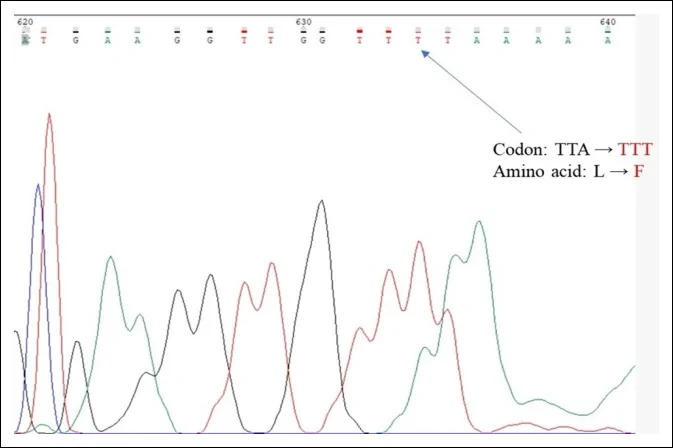
Sequencing chromatogram of feline parvovirus isolate PX365589 showing TTT → F substitution.

**Figure 8:**
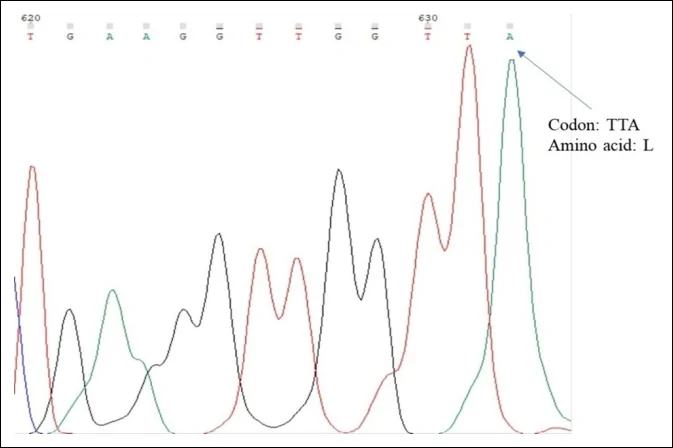
Sequencing chromatogram of feline parvovirus isolate PX365590 showing TTA → L substitution.

**Figure 9:**
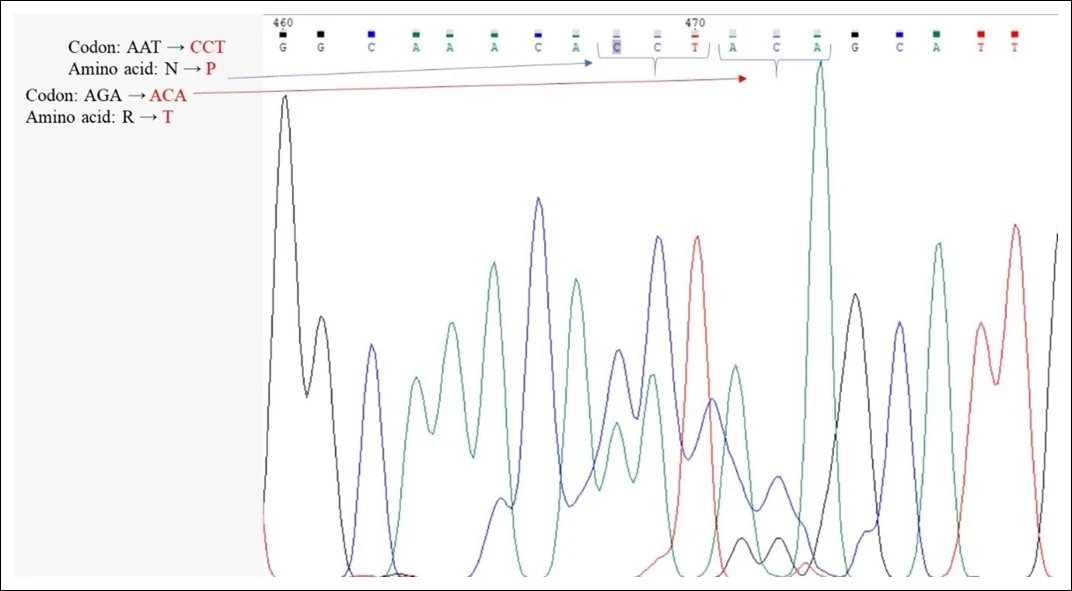
Sequencing chromatogram of canine parvovirus isolate PX365596 showing CCT → P and ACA → T substitutions.

**Figure 10:**
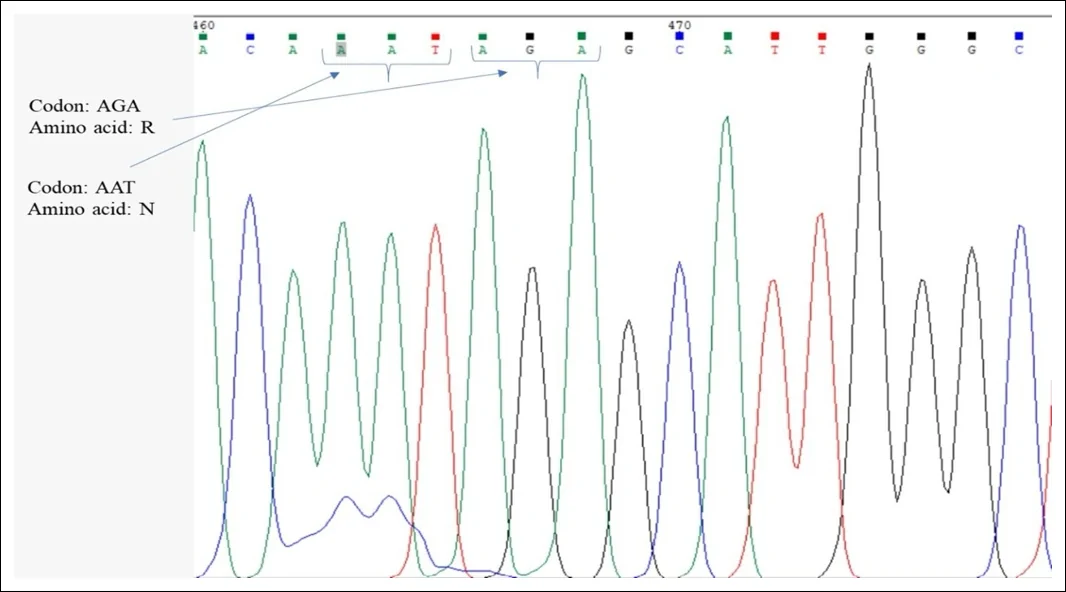
Sequencing chromatogram of canine parvovirus isolate PX365595 showing AAT → N and AGA → R substitutions.

In contrast, CPV isolates showed greater variability and possessed characteristic amino acid signatures consistent with the CPV-2c variant. Phylogenetic analysis clustered the sequences into two distinct groups corresponding to FPV and CPV, with CPV isolates grouping closely with recent CPV-2c strains reported from Asia and Europe. Representative sequencing chromatograms illustrating nucleotide substitutions and corresponding amino acid changes in FPV and CPV isolates are presented in Figures 7–10. Detailed comparison of amino acid substitutions at selected *VP2* loci is presented in Table 3, whereas sequence identity and closest GenBank matches are summarized in Table 4.

**Figure 11:**
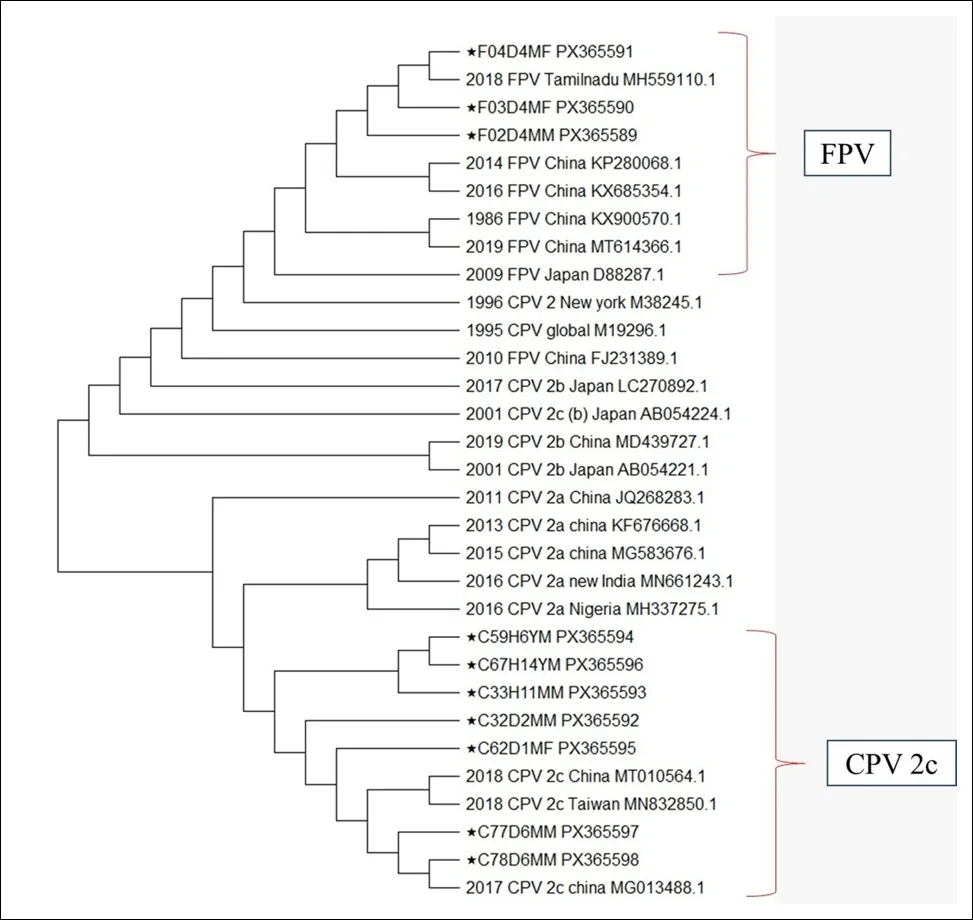
Phylogenetic tree of the *VP2* gene from feline and canine parvovirus isolates, with reference parvovirus sequences. The evolutionary history was inferred using the Maximum Likelihood method and the Tamura three-parameter model.

Phylogenetic analysis of partial *VP2* gene sequences grouped the Gujarat isolates into two distinct clusters corresponding to FPV and CPV. The analysis revealed clustering of the study isolates with reference CPV-2c strains and classical FPV strains. Because only partial *VP2* sequences were analyzed, definitive subtype assignment for all isolates should be interpreted with caution. The study isolates clustered predominantly with contemporary CPV-2c strains while maintaining genetic relatedness to previously reported CPV-2a lineages. Although partial sequences limited definitive subtype classification, the clustering pattern indicated genetic relatedness to contemporary Asian strains. The observed separation from vaccine and prototype strains suggests minor antigenic divergence among circulating parvoviruses (Figure 11). However, the use of partial *VP2* gene sequences limited the ability to achieve complete subtype differentiation and full genomic characterization of circulating parvovirus strains.

**Table 3:** Comparison of amino acid residues at selected loci among feline parvovirus and canine parvovirus type 2 isolates from Gujarat, India, 2025, and reference strains from different regions.

**Isolate ** **details**					**Mutated ** **loci of amino acid**											
**ID**	**Accession No.**	**Subtype**	**Location**	**Year**	**100**	**131**	**135**	**150**	**151**	**165**	**168**	**173**	**191**	**192**	**203**	**206**
F02D4MM	PX365589	FPV	Gujarat, India	2025	V	T	F	N	R	S	A	D	D	Y	E	F
F03D4MF	PX365590	FPV	Gujarat, India	2025	V	T	F	N	R	S	A	D	D	Y	E	L
F04D4MF	PX365591	FPV	Gujarat, India	2025	V	T	F	N	R	S	A	D	D	Y	?	L
1986_FPV_ China	KX900570	FPV	China	1986	V	T	F	N	R	S	A	D	D	Y	?	L
2009_FPV_Japan	D88287	FPV	Japan	2009	I	T	F	N	R	S	A	D	D	Y	?	L
2010_FPV_ China	FJ231389	FPV	China	2010	I	T	F	N	R	S	A	D	N	Y	?	L
2014_FPV_ China	KP280068	FPV	China	2014	V	T	F	N	R	S	A	D	D	Y	?	L
2016_FPV_China	KX685354	FPV	China	2016	V	T	F	N	R	S	A	D	D	Y	?	L
2018_FPV_ Tamil Nadu	MH559110	FPV	Tamil Nadu	2018	V	T	F	N	R	S	A	D	D	Y	?	L
2019_ FPV_China	MT614366	FPV	China	2019	V	T	F	N	R	S	A	D	D	Y	?	L
C32D2MM	PX365592	CPV 2c	Gujarat, India	2025	I	T	Y	N	R	A	G	Y	N	I	E	F
C33H11MM	PX365593	CPV 2c new	Gujarat, India	2025	I	T	Y	N	R	A	G	Y	N	I	?	L
C59H6YM	PX365594	CPV 2c	Gujarat, India	2025	I	I	NS	NS	NS	NS	NS	NS	NS	NS	?	NS
C62D1MF	PX365595	CPV 2c	Gujarat, India	2025	I	T	Y	N	R	A	G	Y	N	I	?	L
C67H14YM	PX365596	CPV 2c new	Gujarat, India	2025	I	T	Y	P	T	A	G	Y	N	I	?	L
C77D6MM	PX365597	CPV 2c	Gujarat, India	2025	I	T	Y	N	R	A	G	Y	N	I	?	L
C78D6MM	PX365598	CPV 2c	Gujarat, India	2025	I	T	Y	N	R	A	G	Y	N	I	?	L
1995_CPV_global_	M19296.1	CPV	Global	1995	I	T	F	N	R	S	A	D	N	Y	?	L
1996_CPV 2_ New York	M38245.1	CPV 2	New York	1996	I	T	F	N	R	S	A	D	N	Y	?	L
2019_CPV 2a _ China	MD439727.1	CPV 2a 2b 2c	China	2019	I	T	F	N	R	A	G	Y	N	Y	?	L
2016_CPV 2a _Nigeria	MH337275.1	CPV 2	Nigeria	2016	I	T	Y	N	R	A	G	Y	N	I	?	L
2011_CPV 2a_ China	JQ268283.1	CPV 2a	China	2011	I	T	F	N	R	A	G	Y	N	I	?	L
2013_CPV 2a_ China	KF676668.1	CPV 2a	China	2013	I	T	Y	N	R	A	G	Y	N	I	?	L
2015_CPV 2a_ China	MG583676.1	CPV 2a	China	2015	I	T	Y	N	R	A	G	Y	N	I	?	L
2001_CPV 2b _ Japan	AB054221.1	CPV 2b	Japan	2001	I	T	F	N	R	A	G	Y	N	Y	?	L
2017_CPV 2b_Japan	LC270892.1	CPV 2b	Japan	2017	I	T	F	N	R	A	V	Y	N	Y	?	L
2017_CPV 2c _China	MG013488.1	CPV 2c	China	2017	I	T	Y	N	R	A	G	Y	N	I	?	L
2018_CPV 2c_China	MT010564.1	CPV 2c	China	2018	I	T	Y	N	R	A	G	Y	N	I	?	L
2018_CPV 2c_Taiwan	MN832850.1	CPV 2c	Taiwan	2018	I	T	Y	N	R	A	G	Y	N	I	?	L
2001_CPV 2c (b)_ Japan	AB054224.1	CPV 2c(b)	Japan	2001	I	T	F	N	R	A	D	Y	N	Y	?	L
2016_CPV 2a new _India	MN661243.1	CPV 2a new	India	2016	I	T	Y	N	R	A	G	Y	N	I	?	L

Green = FPV-specific variation; red = CPV-specific mutation; NS = not sequenced;? = undetermined residue.

### Statistical analysis

Descriptive statistics were used to summarize the prevalence of FPV and CPV infections among cats and dogs. No inferential statistical analysis was performed in the present study.

**Table 4:** Sequence identity and closest GenBank matches of feline parvovirus and canine parvovirus isolates determined by BLASTn analysis.

**Sr. No.**	**Query**	**Accession No.**	**Coverage (%)**	**Identity (%)**	**Closest accession**	**Virus and strain**	**Country**
1	F02D4MM	PX365589	99	99.84	OQ266795.1	Feline panleukopenia virus	Tamil Nadu, India
2	F03D4MF	PX365590	100	99.84	OQ266795.1	Feline panleukopenia virus	Tamil Nadu, India
3	F04D4MF	PX365591	100	100	OQ266795.1	Feline panleukopenia virus	Tamil Nadu, India
4	C32D2MM	PX365592	99	99.84	OR463619.1	*Protoparvovirus carnivoran1* (CPV-2c)	Italy
5	C33H11MM	PX365593	100	100	MW239601.1	*Protoparvovirus carnivoran1* (CPV-2c-new)	Viet Nam: Hai Phong
6	C59H6YM	PX365594	99	100	MT488467.1	CPV-2c	China
7	C62D1MF	PX365595	100	99.84	OR463619.1	*Protoparvovirus carnivoran1* (CPV-2c)	Italy
8	C67H14YM	PX365596	100	99.39	MW239601.1	*Protoparvovirus carnivoran1* (CPV-2c-new)	Viet Nam: Hai Phong
9	C77D6MM	PX365597	100	100	OR296263.1	CPV-2c	India: Chennai, Tamil Nadu
10	C78D6MM	PX365598	100	100	OR463619.1	*Protoparvovirus carnivoran1* (CPV-2c)	Italy

## DISCUSSION

### Sample collection and epidemiological characteristics

The present study provides a comprehensive epidemiological assessment of parvoviral infections in companion animals in Gujarat, India, based on samples collected from diverse sources, including veterinary hospitals, private clinics, rescue organizations, shelters, and apparently healthy animals. Such heterogeneous sampling strengthens the reliability of the findings and provides a broader understanding of viral circulation within the companion animal population. The inclusion of both clinically affected and apparently healthy animals enabled the detection of subclinical infections, which are recognized as important contributors to viral maintenance and transmission within susceptible populations [14, 15].

A higher proportion of animals in this study exhibited clinical signs consistent with gastroenteritis (58.5%), including diarrhea, vomiting, dehydration, and anorexia, which are classical clinical manifestations of FPV and CPV infections [1, 16,17]. However, the detection of infection in apparently healthy animals demonstrates the presence of asymptomatic or subclinical carriers capable of shedding virus into the environment. Such animals may play an important epidemiological role by maintaining viral circulation and facilitating indirect transmission, particularly in high-density environments such as shelters, breeding facilities, and multi-pet households [14,18]. These findings emphasize that clinically healthy animals should not be overlooked in surveillance programs, as they may serve as hidden reservoirs of infection.

Most positive animals were in the 3–6-month age group, consistent with previous reports that young animals are particularly susceptible because maternally derived antibodies gradually decline while the immune system remains immature [2, 16]. During this transitional period, maternal antibodies may decrease below protective concentrations yet remain sufficiently high to interfere with vaccine-induced immunity, thereby creating a period of increased susceptibility to infection [2]. Furthermore, the rapid proliferation of intestinal crypt epithelial cells in young animals provides an ideal environment for parvoviral replication, contributing to severe intestinal damage and clinical disease [1]. These findings reinforce the importance of appropriate vaccination schedules and timely booster immunization in young companion animals.

No apparent association was observed between sex and infection status, suggesting that susceptibility to FPV and CPV is independent of sex. Similar observations have been reported previously, where no consistent sex predisposition was identified among naturally infected companion animals [3, 19]. Breed-related observations indicated relatively higher representation of infections among Labrador Retrievers and German Shepherds. Previous investigators have suggested that this trend may reflect genetic susceptibility, breed-associated immune variability, or management-related factors rather than true breed-specific susceptibility [3]. In contrast, Persian and nondescript cats exhibited comparable infection rates, indicating relatively uniform susceptibility across feline breeds.

Vaccination status appeared to influence infection dynamics. Fully vaccinated cats showed lower FPV detection rates, supporting the protective effectiveness of current vaccination programs [2]. Nevertheless, CPV infection was detected in vaccinated dogs, suggesting possible vaccine failure, incomplete vaccination schedules, interference from maternally derived antibodies, improper vaccine handling, or the circulation of antigenically divergent viral variants that partially escape vaccine-induced immunity [4, 11]. Similar breakthrough infections associated with CPV-2c have been documented in recent studies, emphasizing the need for continuous monitoring of circulating field strains and periodic evaluation of vaccine efficacy [20].

Seasonal variation, characterized by increased positivity during summer and winter, may reflect the exceptional environmental stability of parvoviruses. These viruses can remain infectious for prolonged periods under favorable environmental conditions, thereby facilitating indirect transmission via contaminated fomites and environmental surfaces [14]. Seasonal fluctuations in temperature, humidity, animal movement, and management practices may further influence viral persistence and transmission dynamics [18]. The broad geographical distribution of samples collected from veterinary hospitals, private clinics, and shelters across the study area further demonstrates the endemic nature of parvoviral infections within Gujarat and highlights the widespread circulation of these viruses under different management systems.

Overall, the epidemiological findings of the present study are consistent with previous reports demonstrating that young, unvaccinated, and densely housed companion animals are at greater risk of parvoviral infection [2, 3, 14]. The identification of infection in both clinically affected and apparently healthy animals further emphasizes the importance of routine molecular surveillance, improved vaccination coverage, enhanced biosecurity practices, and early diagnosis to reduce viral transmission and improve disease control in companion animal populations.

### RAT and conventional PCR

RATs are widely used as point-of-care diagnostic tools for parvoviral infections because they are rapid, inexpensive, and suitable for field use. In the present study, however, RAT detected parvoviral antigen in only 5% of samples, which was considerably lower than the detection rates obtained by molecular methods. This finding demonstrates the limited sensitivity of antigen-based assays, particularly when viral loads are low or samples are collected during the early or late stages of infection, when antigen concentrations may fall below the assay detection threshold [10, 15]. Similar observations have been reported previously, in which a substantial proportion of RAT-negative samples were subsequently confirmed positive by PCR or qPCR [11, 18].

The comparatively higher detection rate observed in cats than in dogs using RAT may be related to differences in viral shedding patterns or the stage of infection at sampling. An additional observation was the apparent dual positivity of two canine samples with both FPV and CPV antigen kits, suggesting antigenic cross-reactivity. Because FPV and CPV share more than 98% nucleotide identity within the *VP2* gene, the viruses possess highly similar antigenic epitopes that may be recognized by monoclonal antibodies incorporated into lateral-flow immunoassays [6, 7]. Such cross-reactivity has been documented previously and may complicate interpretation of antigen-based assays when closely related parvoviruses are present [15, 21].

The discrepancy between RAT and molecular assays observed in the present study may also be explained by the relatively high antigen concentration required for visual detection in lateral-flow assays. Samples containing low viral titers, immune-complexed viral antigen, or partially degraded viral proteins may yield false-negative results [10]. Conversely, nonspecific binding or faint reaction lines may occasionally produce false-positive interpretations, thereby reducing diagnostic reliability [18]. These findings indicate that RAT should primarily be regarded as a rapid screening tool rather than a definitive diagnostic assay.

Conventional PCR targeting the *VP2* gene demonstrated a substantially higher detection rate (19.5%) than RAT, confirming its superior analytical sensitivity for detecting parvoviral DNA. Amplification of the expected 681 bp fragment from both feline and canine samples confirmed active circulation of parvoviruses within the study region. These findings are consistent with previous studies demonstrating that PCR reliably detects parvoviral DNA directly from fecal samples, including specimens containing antigen concentrations below the detection limit of rapid tests [5, 10,22].

Nevertheless, the PCR detection rate observed in this study was lower than those reported in several previous investigations from India, where positivity ranged from 28% to 77% among clinically suspected animals [11]. This variation is likely attributable to differences in study design, particularly the inclusion of both clinically healthy and symptomatic animals in the present investigation, as well as differences in sample type, disease stage, viral load, and DNA extraction methodology. Such methodological differences may substantially influence amplification efficiency and diagnostic sensitivity.

A major limitation of conventional PCR is its inability to distinguish FPV from CPV because the primers target highly conserved regions of the *VP2* gene. Similar limitations have been reported previously, where PCR successfully confirmed parvoviral infection but required additional molecular assays for virus typing and differentiation [15, 20]. Detection of parvoviral DNA in both feline and canine samples also supports the co-circulation of FPV and CPV within the study region and raises the possibility of cross-species transmission, which has previously been documented under both natural and experimental conditions [6, 8].

Compared with qPCR, conventional PCR exhibits lower analytical sensitivity. Previous investigations have shown that qPCR can detect as few as 10–100 copies of viral DNA, making it approximately 10–100 times more sensitive than conventional PCR [20]. Consequently, the lower PCR detection rate observed in the present study may reflect reduced analytical sensitivity, particularly in samples containing low viral loads or fecal-derived PCR inhibitors [15]. Despite these limitations, conventional PCR remains a valuable diagnostic tool because of its relatively low cost, accessibility, and ability to confirm parvoviral DNA. However, precise differentiation of FPV and CPV and detailed molecular epidemiological investigations require more sensitive and discriminatory techniques such as probe-based qPCR. Overall, the present findings indicate that although RAT provides convenient field screening and conventional PCR improves diagnostic sensitivity, probe-based qPCR remains essential for accurate diagnosis, molecular surveillance, and comprehensive epidemiological investigations of companion animal parvoviruses.

### Molecular detection and differentiation of FPV and CPV using qPCR

In the present study, probe-based multiplex qPCR targeting virus-specific regions of the *VP2* gene demonstrated a substantially higher detection rate (62%) than conventional PCR (19.5%) and RAT (5%), confirming its superior analytical sensitivity and specificity. Detection of parvoviral infection in 124 of the 200 samples demonstrates active circulation of these viruses among companion animals in Gujarat. CPV (48.5%) was detected considerably more frequently than FPV (13.5%), indicating the predominance of CPV within the study population. Similar observations have been reported previously, where CPV variants exhibited widespread distribution, efficient transmission, and remarkable environmental persistence, facilitating continued circulation among both canine and feline hosts [15, 18, 23]. Recent reports describing the continuing global evolution and dissemination of CPV variants further support these findings [24, 25, 26].

The comparatively lower detection of FPV in cats may reflect improved vaccination coverage and increasing herd immunity within feline populations [2, 17]. Moreover, FPV infections are typically acute and associated with relatively short periods of viral shedding, reducing the probability of detection during cross-sectional investigations [14]. In contrast, CPV exhibits prolonged environmental survival and greater host adaptability, characteristics that likely contribute to its higher prevalence within the present study [1, 7].

An important finding of this investigation was the detection of CPV in feline samples and occasional detection of FPV in canine samples, suggesting possible cross-species transmission. This observation is consistent with previous reports showing that CPV variants readily infect cats, whereas FPV replicates only to a limited extent in dogs [5, 8, 27]. Such host adaptation is largely governed by amino acid substitutions within the *VP2* capsid protein that influence receptor binding and host tropism [6, 7]. Detection of CPV in cats further supports previous reports describing asymptomatic or subclinical CPV infection in feline populations [21].

The superior sensitivity of qPCR compared with conventional PCR observed in this study is attributable to real-time amplification monitoring and fluorescent hydrolysis probes, which permit detection during the exponential phase of amplification. Previous studies have demonstrated that qPCR detects very low viral copy numbers and is substantially more sensitive than conventional PCR [20, 24, 28, 29]. Consequently, additional positive samples with low viral titers were identified by qPCR that would have remained undetected by conventional PCR.

The discrepancies observed between PCR and qPCR in a limited number of samples may be attributed to DNA degradation, amplification inhibitors commonly present in fecal specimens, and variation in primer or probe binding efficiency. Fecal samples frequently contain substances that can inhibit DNA amplification, thereby reducing diagnostic sensitivity and contributing to false-negative PCR results [30]. In addition, differences in sample handling, storage conditions, and nucleic acid extraction procedures may influence assay performance. The incorporation of an internal amplification control into the qPCR assay further enhanced assay reliability by minimizing false-negative results caused by amplification failure or inhibition, consistent with current recommendations for molecular diagnostics [10, 15].

Overall, the present findings demonstrate that probe-based qPCR is a highly sensitive, specific, and reliable method for detecting and differentiating FPV and CPV infections. The predominance of CPV, together with evidence of cross-species transmission, illustrates the dynamic epidemiology of companion animal parvoviruses in Gujarat. These findings emphasize the importance of continuous molecular surveillance, implementation of highly sensitive molecular diagnostic techniques, and regular monitoring of circulating viral variants to support effective disease surveillance, vaccination strategies, and long-term control of parvoviral infections.

### Top of Form

### Bottom of Form

### Virus isolation

Virus isolation remains an important confirmatory method for demonstrating the presence of infectious viral particles and investigating viral replication characteristics. In the present study, successful virus isolation was achieved in four of nine molecularly confirmed parvovirus-positive samples (44.44%) using CRFK cells, as evidenced by characteristic CPE and subsequent molecular confirmation. The observed CPE, including cell rounding, aggregation, granulation, and detachment, are consistent with the well recognized cytopathogenic properties of parvoviruses in susceptible cell cultures [1, 14].

The appearance of CPE between the third and fourth passages indicates that field isolates required an initial adaptation period before efficient replication under *in vitro* conditions. Similar delayed development of CPE has been reported previously, where parvoviral field isolates produced only mild to moderate cytopathic changes following serial passages in susceptible cell lines [31, 32]. This delayed adaptation may result from low viral titers in clinical specimens, partial degradation of viral particles, or the requirement for adaptation to artificial cell culture conditions.

The relatively low virus isolation rate observed in this study may be explained by several factors, including sample quality, viral load, stage of infection, and the presence of inhibitory substances or neutralizing antibodies within fecal material. Successful virus isolation depends on both the integrity of infectious viral particles and the susceptibility of the host cell line used [33]. Moreover, inclusion of both clinically healthy and clinically affected animals likely introduced variation in viral loads among specimens, thereby influencing isolation efficiency.

Confirmation of parvoviral DNA in CPE-positive cultures by PCR during the early passages verified active viral replication in CRFK cells. However, the absence of detectable amplification in later passages, despite initial PCR positivity, suggests reduced viral replication or progressive loss of detectable viral nucleic acid during serial passage. This phenomenon may reflect reduced viral fitness following repeated passaging, degradation of viral nucleic acids due to repeated freeze-thaw cycles, or suboptimal culture conditions that affect viral propagation [30]. Additionally, accumulation of defective or noninfectious viral particles during serial passage may further contribute to declining viral loads and subsequent PCR negativity.

Another possible explanation for the reduction in detectable viral DNA during later passages is activation of cellular antiviral defense mechanisms that suppress viral replication under *in vitro* conditions. Continuous cell culture may also exert selective pressure on field strains, resulting in viral attenuation or progressive loss of infectivity over time [7]. These findings emphasize the importance of confirming viral replication during the early passages when virus recovery is most successful.

The successful propagation of parvoviruses in CRFK cells observed in the present study supports previous reports that both FPV and CPV replicate efficiently in feline-derived cell lines. The broad host range of carnivore parvoviruses is largely determined by amino acid substitutions within the *VP2* capsid protein that influence receptor binding and host tropism [6, 7]. Successful isolation of viruses from both feline and canine specimens using CRFK cells therefore further supports the concept of shared cellular tropism and receptor utilization between FPV and CPV strains.

Although alternative cell lines, including A-72 and Madin-Darby canine kidney (MDCK) cells, have been reported to exhibit greater sensitivity for parvovirus isolation, CRFK cells remain a reliable and widely accepted system for propagation of both FPV and CPV [33]. The findings of the present study reinforce the usefulness of CRFK cells while highlighting the challenges of recovering field isolates, particularly those with low viral loads or reduced viral viability.

Overall, the present study demonstrates that although virus isolation provides valuable information regarding viral infectivity and biological behavior, it is considerably less sensitive than molecular diagnostic methods such as PCR and qPCR. Combining cell culture with molecular confirmation remains essential for comprehensive characterization of circulating parvoviruses. The relatively low isolation efficiency observed in this investigation further emphasizes the importance of optimized sample handling, early passage monitoring, and integration of sensitive molecular assays to maximize virus recovery and diagnostic accuracy. These findings confirm that virus isolation serves primarily as a confirmatory method and is highly dependent on sample quality and viral viability compared with molecular diagnostic approaches.

### Molecular characterization and phylogenetic analysis of FPV and CPV

Molecular characterization based on the *VP2* gene provides valuable insights into parvovirus evolution, host adaptation, and antigenic diversity. In the present study, sequencing of the partial *VP2* gene (approximately 681 bp) from 10 representative isolates confirmed the co-circulation of FPV and CPV among companion animals in Gujarat, India. Successful amplification and sequencing of all selected isolates further demonstrate that the *VP2* gene remains a robust molecular marker for epidemiological investigations and evolutionary studies of carnivore parvoviruses [12, 20].

The FPV isolates identified in this study exhibited a high degree of nucleotide conservation with only minor amino acid substitutions compared with reference strains. Conserved amino acid residues at positions 131, 135, 150, 151, and 165 indicate considerable genetic stability among circulating FPV strains. These findings agree with previous reports indicating that FPV evolves more slowly than CPV and maintains remarkable genomic conservation across geographically distinct populations [1, 14,34,26]. Minor substitutions observed at positions 203 and 206 likely represent localized genetic drift and are unlikely to substantially influence viral antigenicity or host range.

In contrast, CPV isolates exhibited greater genetic variability, with multiple amino acid substitutions at key antigenic sites in the *VP2* protein. Characteristic substitutions at positions 135 (F→Y), 165 (S→A), and 168 (A→G) are consistent with CPV-2c variant lineages. These mutations have previously been associated with alterations in viral antigenicity, receptor binding, host adaptation, and viral fitness, contributing to the successful global dissemination of CPV variants [4, 20, 35]. Most isolates displayed amino acid profiles comparable to those reported previously from India and neighboring Asian countries, indicating continued regional circulation of established CPV lineages.

One isolate possessed unique substitutions at positions 150 and 151, suggesting possible regional adaptation or emergence of a novel viral variant. Amino acid substitutions within the *VP2* protein are of particular biological importance because even a single substitution may influence viral fitness, antigenicity, host range, and vaccine efficacy [7]. Consequently, these observations support the occurrence of ongoing microevolution of CPV under field conditions and further emphasize the importance of continuous molecular surveillance.

BLASTn analysis demonstrated high nucleotide identity (99%–100%) between the FPV isolates and previously reported strains from India and other Asian countries. Similarly, CPV isolates exhibited high sequence similarity with strains reported from Europe and Southeast Asia [26, 36]. These findings suggest that circulating field strains share a common evolutionary ancestry and that international movement of companion animals, together with the exceptional environmental stability of parvoviruses, may facilitate global dissemination of CPV variants [18].

Phylogenetic analysis based on partial *VP2* gene sequences clearly separated the study isolates into two principal clusters corresponding to FPV and CPV, confirming their distinct evolutionary relationships. Within the CPV cluster, study isolates grouped closely with CPV-2a and CPV-2c variants while remaining distinct from prototype and vaccine strains. This clustering pattern indicates that circulating CPV strains in Gujarat are genetically related to contemporary Asian variants while continuing to undergo gradual evolutionary divergence. Similar phylogenetic relationships have recently been reported from several countries, where CPV variants continue to evolve while maintaining conserved genetic characteristics [20, 29].

The observed phylogenetic separation of field isolates from vaccine and prototype strains suggests minor antigenic divergence that may affect vaccine effectiveness under field conditions, as reported previously [26, 29]. Recent investigations have further demonstrated the increasing predominance of CPV-2c over earlier variants such as CPV-2a in several countries, including India [29]. In addition, accumulating evidence suggests that antigenic variation among circulating CPV strains may contribute to vaccine breakthrough infections, particularly in puppies with incomplete or waning immunity [37].

The inability to evaluate several important antigenic residues, including positions 297 and 426, due to the availability of only partial *VP2* sequences represents a limitation of the present study. Nevertheless, the overall amino acid substitution pattern together with phylogenetic clustering strongly supports the circulation of CPV-2c-like variants in Gujarat. The observed genetic distance between field isolates and vaccine strains further indicates ongoing antigenic divergence, warranting continued molecular monitoring and evaluation of vaccine performance.

Detection of both FPV and CPV in the present investigation, together with their distinct phylogenetic clustering, reinforces the concept of co-circulation and possible cross-species transmission among companion animals [28]. CPV variants are well recognized for their ability to infect feline hosts, whereas FPV demonstrates only limited adaptation to dogs, highlighting the critical role of *VP2* mutations in determining host specificity [6, 8]. Detection of CPV in feline samples within the present study further supports the expanding host range of contemporary CPV variants.

Overall, the molecular characterization performed in this study demonstrates that FPV remains genetically stable, whereas CPV continues to undergo adaptive evolution through the emergence of variant strains. The coexistence of conserved and variable regions within the *VP2* gene illustrates the balance between structural conservation and evolutionary flexibility. These findings emphasize the importance of continuous genomic surveillance to identify emerging mutations, monitor viral evolution, and evaluate their potential influence on diagnostics and vaccine efficacy.

The present study has several limitations. Partial sequencing of the *VP2* gene restricted comprehensive subtype characterization of all isolates. Furthermore, the absence of inferential statistical analyses prevented detailed evaluation of epidemiological risk factors associated with infection. Future investigations incorporating whole-genome sequencing, larger sample sizes, and comprehensive epidemiological datasets will provide a more complete understanding of viral evolution, transmission dynamics, and host adaptation.

The present investigation provides important baseline molecular and epidemiological information regarding the circulation of FPV and CPV in Gujarat, India. The findings demonstrate the co-circulation of genetically distinct yet closely related parvoviruses, evidence of cross-species transmission, and the continued evolution of CPV variants. Collectively, these observations highlight the need for sustained molecular surveillance, optimization of vaccination strategies, and implementation of highly sensitive diagnostic methods such as qPCR to strengthen disease surveillance and improve long-term control of parvoviral infections, particularly in regions with large populations of stray and unvaccinated companion animals.

## CONCLUSION

This study provides a comprehensive molecular epidemiological investigation of FPV and CPV infections among companion animals in Gujarat, India. The findings demonstrated widespread circulation of parvoviruses, with qPCR identifying a substantially higher prevalence (62.0%) than conventional PCR (19.5%) and RAT (5.0%), confirming the superior diagnostic sensitivity of probe-based qPCR. Molecular characterization based on the *VP2* gene revealed that FPV isolates remained highly conserved, whereas CPV isolates exhibited greater genetic diversity, predominantly clustering with contemporary CPV-2c variants. Detection of CPV in feline samples and occasional detection of FPV in canine samples further indicated the potential for cross-species transmission, while successful virus isolation in CRFK cells confirmed the infectivity of circulating field strains.

From a practical perspective, the study highlights the importance of implementing highly sensitive molecular diagnostic methods, particularly qPCR, for routine diagnosis, surveillance, and differentiation of FPV and CPV infections. The predominance of CPV-2c-like variants and evidence of antigenic divergence from prototype and vaccine strains emphasize the need for continuous molecular surveillance and periodic evaluation of vaccine effectiveness. These findings also reinforce the importance of maintaining adequate vaccination coverage, strengthening biosecurity practices, and monitoring apparently healthy animals that may serve as asymptomatic carriers contributing to viral transmission.

A major strength of this study is the integration of epidemiological investigation, comparative evaluation of diagnostic methods, virus isolation, molecular characterization, and phylogenetic analysis within a single regional survey. Inclusion of both clinically affected and apparently healthy companion animals from diverse management systems provides a comprehensive overview of parvovirus circulation under field conditions.

Nevertheless, the study has certain limitations. Partial sequencing of the *VP2* gene limited complete subtype characterization and assessment of all antigenically important mutations. In addition, the cross-sectional study design and reliance on descriptive statistics precluded detailed evaluation of epidemiological risk factors and temporal transmission dynamics.

Future investigations should incorporate whole-genome sequencing, longitudinal surveillance, larger multicenter sample collections, and advanced epidemiological analyses to better understand viral evolution, host adaptation, transmission pathways, and the emergence of novel variants. Continuous monitoring of circulating strains together with regular assessment of vaccine efficacy will be essential to ensure effective prevention and control of parvoviral infections.

In conclusion, the present study provides important baseline epidemiological and molecular evidence demonstrating the co-circulation of genetically distinct FPV and CPV strains in Gujarat, India. The predominance of CPV-2c-like variants, evidence of cross-species transmission, and superior performance of qPCR underscore the necessity for continuous molecular surveillance, sensitive diagnostic strategies, and optimized vaccination programs to improve the control of parvoviral infections in companion animal populations.

## DATA AVAILABILITY

All data generated or analyzed during this study are included in this published article. The nucleotide sequences generated during this study have been deposited in the GenBank database under accession numbers PX365589–PX365598.

## GENERATIVE AI DECLARATION

The authors declare that generative artificial intelligence (AI) tools were used solely to improve language, grammar, and readability during manuscript preparation. All scientific content, data analysis, interpretation of results, and conclusions were developed and verified by the authors. The authors take full responsibility for the accuracy, integrity, and originality of the work presented, and no AI tool was listed as an author.

## AUTHORS’ CONTRIBUTIONS

FAP: Conceptualization, sample collection, laboratory investigation, molecular analysis, data curation, formal analysis, writing of the original draft, and manuscript preparation. AP: Study supervision, research planning, methodology development, assay standardization, data interpretation, manuscript review, and editing. NMR and SPA: Laboratory investigations, sample processing, data collection, manuscript review, and editing. PGK: Technical guidance, assay standardization, molecular analysis support, and manuscript review and editing. RAM and VRN: Assay standardization support and manuscript review and editing. ASP: Sample collection, laboratory assistance, and technical support. All authors have read and approved the final version of the manuscript.

## References

[1] Parrish CR (1995). Pathogenesis of feline panleukopenia virus and canine parvovirus. Baillieres Clin Haematol.

[2] Jakel V, Cussler K, Hanschmann KM, Truyen U, König M, Kamphuis E (2012). Vaccination against feline panleukopenia: implications from a field study in kittens. BMC Vet Res.

[3] Chisty NN, Belgrad JP, Al Sattar A, Akter S, Hoque MA (2020). Clinico-epidemiological investigation of feline panleukopenia and parvoviral enteritis in the two largest pet hospitals in Bangladesh. J Adv Vet Anim Res.

[4] Truyen U (2006). Evolution of canine parvovirus—a need for new vaccines? Vet Microbiol.

[5] Mochizuki M, Horiuchi M, Hiragi H, San Gabriel MC, Yasuda N, Uno T (1996). Isolation of canine parvovirus from a cat manifesting clinical signs of feline panleukopenia. J Clin Microbiol.

[6] Truyen U, Gruenberg A, Chang SF, Obermaier B, Veijalainen P, Parrish CR (1995). Evolution of the feline-subgroup parvoviruses and the control of canine host range in vivo. J Virol.

[7] Hueffer K, Parrish CR (2003). Parvovirus host range, cell tropism and evolution. Curr Opin Microbiol.

[8] Ikeda Y, Nakamura K, Miyazawa T, Tohya Y, Takahashi E, Mikami T (2002). Feline host range of canine parvovirus: recent emergence of new antigenic types in cats. J Clin Microbiol.

[9] Battilani M, Balboni A, Giunti M, Prosperi S, Cozzi M (2013). Genetic diversity of feline parvovirus: implications for molecular epidemiology. Infect Genet Evol.

[10] Jacobson LS, Clark C, Thompson PN (2021). Diagnosis of canine parvovirus infection: a comparison of diagnostic tests. J Vet Diagn Invest.

[11] Kadam P, Rana N, Patel A, Koringa P (2024). Molecular detection and characterization of parvovirus infections in companion animals in India. Indian J Vet Res.

[12] Carreño A, Van Brussel K, Martella V (2021). Development of molecular assays targeting the VP2 gene for detection and differentiation of feline and canine parvoviruses. Vet Res Commun.

[13] Decaro N, Desario C, Lucente MS, Amorisco F, Campolo M, Elia G (2008). Specific identification of feline panleukopenia virus and its rapid differentiation from canine parvoviruses using minor groove binder probes. J Virol Methods.

[14] Stuetzer B, Hartmann K (2014). Feline parvovirus infection and associated diseases. Vet J.

[15] Decaro N, Buonavoglia C (2012). Canine parvovirus—A review of epidemiological and diagnostic aspects. Vet Microbiol.

[16] Trotman TK (2014). Feline panleukopenia: clinical signs, diagnosis, and treatment. J Feline Med Surg.

[17] Sykes JE, Sykes JE Feline panleukopenia virus infection. Canine and Feline Infectious Diseases. Elsevier; 2013. p. 187–194.

[18] Miranda C, Thompson G (2016). Canine parvovirus: the worldwide occurrence of antigenic variants. J Gen Virol.

[19] Kalli I, Leontides LS, Mylonakis ME, Adamama-Moraitou K, Rallis T, Koutinas AF (2010). Factors affecting occurrence, duration of hospitalization, and outcome in canine parvovirus infection. Res Vet Sci.

[20] Streck AF, Canal CW, Truyen U (2013). Molecular epidemiology and evolution of canine parvovirus. Infect Genet Evol.

[21] Clegg SR, Coyne KP, Dawson S, Spibey N, Gaskell RM, Radford AD (2012). Canine parvovirus in asymptomatic feline carriers. Vet Microbiol.

[22] Garcia RCN, Castro TX, Miranda SC, Labarthe NV, Silva LE (2011). Detection of canine parvovirus in diarrheic dogs from Brazil. Braz J Microbiol.

[23] Diakoudi G, Lanave G, Capozza P, Di Profio F, Melegari I, Marsilio F (2022). Molecular epidemiology and evolution of canine parvovirus strains circulating in Europe. Transbound Emerg Dis.

[24] Xue C, Zhu Y, Zhang X, Wang J, Li Y (2023). Advances in nanoPCR and quantitative PCR for detection of animal parvoviruses. Front Vet Sci.

[25] Raja P, Sorna Mallika K, Yuvachandran Viva V, Parthiban M, Sathish G, Vinitha V (2024). Complete genome sequence and phylogenetic analysis of feline panleukopenia virus from India. VirusDisease.

[26] Wang Z, Jiang Y, Xin T, Yuan W, Guo X, Zhou H (2024). Genetic characterization and evolutionary analysis of canine parvovirus in Tangshan, China. Transbound Emerg Dis.

[27] Hoang M, Wu HY, Lin WH (2020). Limited replication of feline panleukopenia virus in canine hosts: experimental insights. Vet Microbiol.

[28] Maganga GD, Labouba I, Milendz Ikapi SZ, Nkili-Meyong AA, Ngonga Dikongo AM, Boundenga L (2024). Molecular characterization of canine parvovirus variants CPV-2a and CPV-2c associated with vaccinated dogs at Libreville, Gabon. Front Vet Sci.

[29] Reddy H, Srinivas MV, Vasu J, Prabavathy A, Dhodapkar R, Mukhopadhyay HK (2024). Whole-genome sequence analysis of canine parvovirus reveals replacement with a novel CPV-2c strain throughout India. Arch Virol.

[30] Duarte MD, Henriques AM, Barros SC, Fagulha T, Ramos F, Luís T (2012). Comparison of two protocols for DNA extraction from faecal samples for canine parvovirus detection. Vet Microbiol.

[31] Parthiban S, Mukhopadhyay HK, Antony PX, Pillai RM (2011). Isolation and characterization of canine parvovirus in cell culture. Indian J Virol.

[32] Mukhopadhyay HK, Pillai RM, Antony PX, Thanislass J, Srinivasan VA (2017). Molecular and biological characterization of parvovirus isolates. VirusDisease.

[33] Brindhalakshmi B, Mukhopadhyay HK, Thanislass J, Pillai RM, Vijayarani K (2016). Isolation and molecular characterization of canine parvovirus. Vet World.

[34] Van Vuuren M, Steinel A, Goosen WJ, Truyen U (2000). Genetic characterization of feline panleukopenia virus strains from South Africa. J Gen Virol.

[35] Fang N, Li M, Zhang A, Wen L, Li H, Ye X (2025). Cross-species characterization of feline parvovirus and feline-origin canine parvovirus in Southern China (2023–2024): insights into epidemiology, genetic evolution and recombination. BMC Vet Res.

[36] Yang S, Wang S, Feng H (2022). Molecular evolution of feline panleukopenia virus in Asia. Vet Microbiol.

[37] Safwat MS, Khodeir MH, Etman RH (2026). Canine parvovirus type 2 infection in vaccinated puppies: role of vaccination practices and viral antigenic variation. BMC Vet Res.

